# Phenome-wide heritability analysis of the UK Biobank

**DOI:** 10.1371/journal.pgen.1006711

**Published:** 2017-04-07

**Authors:** Tian Ge, Chia-Yen Chen, Benjamin M. Neale, Mert R. Sabuncu, Jordan W. Smoller

**Affiliations:** 1 Athinoula A. Martinos Center for Biomedical Imaging, Massachusetts General Hospital / Harvard Medical School, Charlestown, MA, United States of America; 2 Psychiatric and Neurodevelopmental Genetics Unit, Center for Genomic Medicine, Massachusetts General Hospital, Boston, MA, United States of America; 3 Stanley Center for Psychiatric Research, Broad Institute of MIT and Harvard, Cambridge, MA, United States of America; 4 Analytic and Translational Genetics Unit, Center for Genomic Medicine, Massachusetts General Hospital, Boston, MA, United States of America; 5 School of Electrical and Computer Engineering and Meinig School of Biomedical Engineering, Cornell University, Ithaca, NY, United States of America; Stanford University, UNITED STATES

## Abstract

Heritability estimation provides important information about the relative contribution of genetic and environmental factors to phenotypic variation, and provides an upper bound for the utility of genetic risk prediction models. Recent technological and statistical advances have enabled the estimation of additive heritability attributable to common genetic variants (SNP heritability) across a broad phenotypic spectrum. Here, we present a computationally and memory efficient heritability estimation method that can handle large sample sizes, and report the SNP heritability for 551 complex traits derived from the interim data release (152,736 subjects) of the large-scale, population-based UK Biobank, comprising both quantitative phenotypes and disease codes. We demonstrate that common genetic variation contributes to a broad array of quantitative traits and human diseases in the UK population, and identify phenotypes whose heritability is moderated by age (e.g., a majority of physical measures including height and body mass index), sex (e.g., blood pressure related traits) and socioeconomic status (education). Our study represents the first comprehensive phenome-wide heritability analysis in the UK Biobank, and underscores the importance of considering population characteristics in interpreting heritability.

## Author summary

Heritability of a trait refers to the proportion of phenotypic variation that is due to genetic variation among individuals. It provides important information about the genetic basis of complex traits and indicates whether a phenotype is an appropriate target for more specific statistical and molecular genetic analyses. Recent studies have leveraged the increasingly ubiquitous genome-wide data and documented the heritability attributable to common genetic variation captured by genotyping microarrays for a wide range of human traits. However, heritability is not a fixed property of a phenotype and can vary with population-specific differences in the genetic background and environmental variation. Here, using a computationally and memory efficient heritability estimation method, we report the heritability for a large number of traits derived from the large-scale, population-based UK Biobank, and, for the first time, demonstrate the moderating effect of three major demographic variables (age, sex and socioeconomic status) on heritability estimates derived from genome-wide common genetic variation. Our study represents the first comprehensive heritability analysis across the phenotypic spectrum in the UK Biobank.

## Introduction

The heritability of a trait refers to the proportion of phenotypic variance that is attributable to genetic variation among individuals. Heritability is commonly measured as either the contribution of total genetic variation (broad-sense heritability, *H*^2^), or the fraction due to additive genetic variation (narrow-sense heritability, *h*^2^) [1]. A large body of evidence from twin studies has documented that essentially all human complex traits are heritable. For example, a recent meta-analysis of virtually all twin studies published between 1958 and 2012, encompassing 17,804 traits, reported that the overall narrow-sense heritability estimate across all human traits was 49%, although estimates varied widely across phenotypic domains [2]. Over the past decade, the availability of genome-wide genotyping has enabled the direct estimation of additive heritability attributable to common genetic variation (“SNP heritability” or $h_{SNP}^{2}$) [3–5]. These estimates do not capture non-additive genetic effects such as dominance or epistasis, and provide a lower bound for narrow-sense heritability because they also do not capture contributions (e.g., from rare variants) that are not assayed by most genotyping microarrays and are not well tagged by genotyped variants. Nevertheless, estimates of SNP heritability can provide important information about the genetic basis of complex traits such as the proportion of phenotypic variation that could be explained by common-variant genome-wide association studies (GWAS).

However, heritability is not a fixed property of a phenotype but depends on the population in which it is estimated. As a ratio of variances, it can vary with population-specific differences in both genetic background and environmental variation [1]. For example, twin data have documented variations in the heritability of childhood IQ by socioeconomic status (SES) [6], highlighting that different environment may have different relative contributions to the variance of a phenotype. In addition, heritability estimates for a range of complex phenotypes have been shown to vary according to the sex and age distributions of the sampled populations [2]. Identifying variables that may affect the heritability of complex traits has implications for the design of GWAS, highlighting subgroups and environmental conditions in which common-variant contributions may be diminished or magnified. To date, however, studies of complex trait heritability and the effect of modifying variables have produced mixed results likely due to sample size limitations and population-specific differences in genetic and environmental variance that may be operating in different cohorts.

The UK Biobank (http://www.ukbiobank.ac.uk) provides a unique opportunity to estimate the heritability of traits across a broad phenotypic spectrum in a single population sample. The UK Biobank is a large prospective population-based cohort study that enrolled 500,000 participants aged 40–69 years between 2006 and 2010 [7]. The study has collected a wealth of phenotypic data from questionnaires, physical and biological measurements, and electronic health records as well as genome-wide genotype data. However, this rich data source also presents analytic challenges. For example, with the large sample size, existing heritability estimation methods such as genome-wide complex trait analysis (GCTA) [3–5] and LD (linkage disequilibrium) score regression [8] become computationally expensive and memory intensive, and thus can be difficult to apply. Here we implemented a computationally and memory efficient approach to estimate the heritability for 551 complex traits derived from the interim data release (152,736 subjects) of the UK Biobank, comprising both quantitative phenotypes and disease categories. We then examined how heritability estimates are modified by three major demographic variables: age, sex and socioeconomic status (SES). Our results underscore the importance of considering population characteristics in estimating and interpreting heritability, and may inform efforts to apply genetic risk prediction models for a broad range of human phenotypes.

## Results

We report the heritability for 551 traits that were made available to us through the interim data release of the UK Biobank (downloaded on Mar 3, 2016) and had sufficient sample sizes to achieve accurate heritability estimates (standard error of the heritability estimate smaller than 0.1; 15 disease codes excluded) using a computationally and memory efficient heritability estimation method (see Methods and S1 Text).

**Fig 1:**
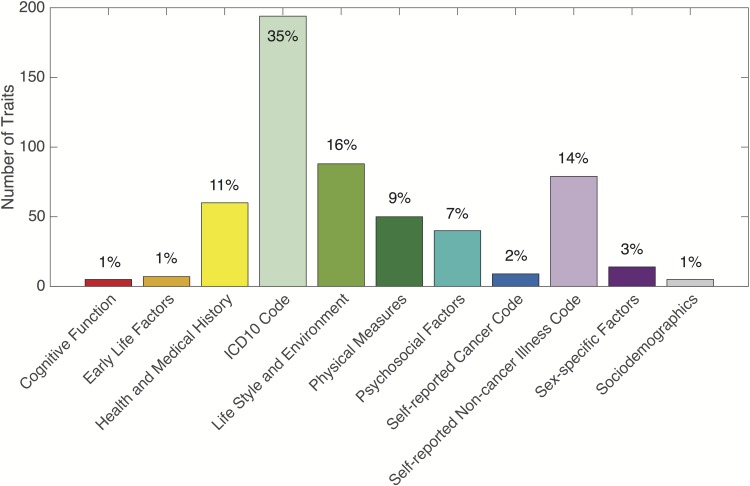
The number of traits in each of the 11 phenotypic domains that make up the 551 traits analyzed in the UK Biobank: cognitive function (5 traits), early life factors (7 traits), health and medical history (60 traits), hospital in-patient main diagnosis ICD-10 codes (194 traits), life style and environment (88 traits), physical measures (50 traits), psychosocial factors (40 traits), self-reported cancer codes (9 traits), self-reported non-cancer illness codes (79 traits), sex-specific factors (14 traits), and sociodemographics (5 traits).

The 551 traits can be classified into 11 general phenotypic domains as defined by the UK Biobank to group individual data fields into broadly related sets: cognitive function (5 traits), early life factors (7 traits), health and medical history (60 traits), hospital in-patient main diagnosis ICD-10 codes (194 traits), life style and environment (88 traits), physical measures (50 traits), psychosocial factors (40 traits), self-reported cancer codes (9 traits), self-reported non-cancer illness codes (79 traits), sex-specific factors (14 traits), and sociodemographics (5 traits). ICD-10 (the International Classification of Diseases, version-10) is a medical classification list published by the World Health Organization (WHO), which contains thousands of diagnostic codes. Fig 1 shows the percentage of each domain that makes up the 551 traits we analyzed. Using the top-level categories and chapters of the self-reported disease and ICD-10 coding tree, we can further break down self-reported non-cancer illness codes and ICD-10 codes into different functional domains (S1 Fig). We note that since we only analyzed disease codes that had prevalence greater than 1% in the sample, distribution of the disease traits across functional domains was skewed. For example, we investigated a large number of gastrointestinal and musculoskeletal traits, while diseases that have low prevalence in the sampled population such as psychiatric disorders were not well represented.

Table 1 lists the top heritable traits in each domain (the most heritable trait and traits with heritability estimates greater than 0.30). S1 Table and S2 Table show the heritability estimates, standard error estimates, sample sizes, covariates adjusted, prevalence in the sample (for binary traits) and other relevant information for all the traits we analyzed. Common genetic variants appear to have an influence on most traits we investigated, although heritability estimates showed heterogeneity within and across trait domains. Complex traits that exhibited high SNP heritability (larger than 0.40) included human height (0.685+/-0.004), skin color (very fair/fair vs. other, 0.556+/-0.008), ease of skin tanning (very/moderately tanned vs. mildly/occasionally/never tanned, 0.454+/-0.006), comparative height at age 10 (taller than average, 0.439+/-0.007; shorter than average, 0.405+/-0.008), rheumatoid arthritis (0.821+/-0.046), hypothyroidism/myxedema (0.814+/-0.017), malignant neoplasm of prostate (0.426+/-0.093), and diabetes diagnosed by doctor (0.414+/-0.016), among others. On the other end of the spectrum, traits such as duration of walks/moderate activity/vigorous activity, frequency of stair climbing, ever had stillbirth, spontaneous miscarriage or termination, painful gums, stomach disorder, fracture, injuries to the head/knee/leg, and pain in joint had zero or close to zero heritability estimates, indicating that their phenotypic variation is largely determined by environmental factors, or there is widespread heterogeneity or substantial measurement error in these phenotypes. SNP heritability estimates for several phenotypes, including diseases with known immune-mediated pathogenesis (rheumatoid arthritis, psoriasis, diabetes, hypothyroidism), were markedly reduced when the major histocompatibility complex (MHC) region was excluded from analysis (S4 Table), and thus need to be interpreted with caution (see Discussion).

**Table 1:** The SNP heritability estimates (h2), standard error (SE) estimates, sample sizes (N), and prevalence (prev) in the sample (for binary traits) for the top heritable traits in each phenotypic domain. All heritability estimates adjusted for genotyping array, UK Biobank assessment center, age at recruitment, sex (except male-specific traits indicated by *) and top 10 principal components of the genotype data as covariates. ^♯^: additionally adjusted for weight; ^§^: additionally adjusted for negative experience. See S1 Table and S2 Table for the way we binarized categorical variables and other relevant information.

Field ID	Field Name	Type	N	Prev	h2	SE
20016	Fluid intelligence score	continuous	34491	NA	0.233	0.011
1697	Comparative height at age 10	categorical	106497	25.36%	0.439	0.007
2976	Age diabetes diagnosed	continuous	5369	NA	1.000	0.074
6148	Eye problems/disorders	categorical	33750	2.04%	0.722	0.097
2443	Diabetes diagnosed by doctor	binary	107935	5.25%	0.414	0.016
6152	Blood clot, DVT, bronchitis, emphysema, asthma, rhinitis, eczema, allergy diagnosed by doctor	categorical	108022	12.37%	0.344	0.010
41202	C61 Malignant neoplasm of prostate	binary	32780	2.24%	0.426*	0.093
41202	C60-C63 Malignant neoplasms of male genital organs	binary	32780	2.46%	0.322*	0.087
1747	Hair colour (natural, before greying)	categorical	107947	4.62%	1.000	0.018
1717	Skin colour	categorical	106773	80.09%	0.556	0.008
1727	Ease of skin tanning	categorical	105966	61.13%	0.454	0.006
6144	Never eat eggs, dairy, wheat, sugar	categorical	107832	2.68%	0.691	0.026
50	Standing height	continuous	107976	NA	0.685	0.004
23105	Basal metabolic rate	continuous	106311	NA	0.397	0.004
23102	Whole body water mass	continuous	106252	NA	0.331^♯^	0.004
23130	Trunk predicted mass	continuous	106157	NA	0.325^♯^	0.004
23106	Impedance of whole body	continuous	106304	NA	0.307	0.004
20151	Forced vital capacity (FVC), best measure	continuous	84301	NA	0.339	0.005
20150	Forced expiratory volume in 1-second (FEV1), best measure	continuous	84305	NA	0.324	0.005
3148	Heel bone mineral density (BMD)	continuous	62546	NA	0.327	0.006
20126	Bipolar and major depression status	categorical	25571	19.91%	0.195^§^	0.032
20001	Prostate cancer	binary	50997	1.59%	0.310*	0.077
20002	Psoriasis	binary	108148	1.15%	1.000	0.047
20002	Thyroid problem (not cancer)	binary	108148	5.77%	0.876	0.015
20002	Rheumatoid arthritis	binary	108148	1.18%	0.821	0.046
20002	Hypothyroidism/Myxoedema	binary	108148	4.83%	0.814	0.017
20002	Diabetes	binary	108148	5.14%	0.396	0.016
20002	Dermatology	binary	108148	4.27%	0.368	0.019
20002	Asthma	binary	108148	12.44%	0.340	0.010
2395	Hair/Balding pattern	categorical	50660	18.51%	0.479*	0.017
2375	Relative age of first facial hair	categorical	49384	6.64%	0.303*	0.030
6138	Qualifications	categorical	107158	30.16%	0.294	0.007

A substantial fraction of the phenotypes we examined were based on self-report illness codes or diagnostic (ICD-10) codes, which may be noisy and have low specificity. However, the SNP heritability estimates for 14 pairs of self-reported illness and ICD-10 codes that represent the same or closely matched diseases were largely consistent and had a Pearson correlation of 0.78 (Table 2), indicating that both phenotypic approaches captured useful and comparable variations in these phenotypes.

**Table 2:** A head-to-head comparison of SNP heritability estimates (h2) for the self-reported illness codes and ICD-10 codes that represent the same or closely matched diseases.

Self-reported disease	h2 (self-reported)	ICD-10 code	h2 (ICD-10)
Breast cancer	0.133	C50 Malignant neoplasm of breast	0.207
Skin cancer	0.061	C43-C44 Melanoma and other malignant neoplasms of skin	0.160
Male genital tract cancer	0.226	C60-C63 Malignant neoplasms of male genital organs	0.322
Prostate cancer	0.310	C61 Malignant neoplasm of prostate	0.426
Cerebrovascular disease	0.066	I60-I69 Cerebrovascular diseases	0.197
Angina	0.176	I20 Angina pectoris	0.136
Liver/Biliary/Pancreas problem	0.074	K80-K87 Disorders of gallbladder, biliary tract and pancreas	0.099
Heart attack/Myocardial infarction	0.184	I21 Acute myocardial infarction	0.193
Gastro-oesophageal reflux (gord)/Gastric reflux	0.099	K21 Gastro-oesophageal reflux disease	0.105
Cholelithiasis/Gall stones	0.123	K80 Cholelithiasis	0.143
Uterine fibroids	0.087	D25 Leiomyoma of uterus	0.054
Enlarged prostate	0.157	N40 Hyperplasia of prostate	0.127
Pneumonia	0.075	J18 Pneumonia—organism unspecified	0.160
Diverticular disease/Diverticulitis	0.195	K57 Diverticular disease of intestine	0.179

**Fig 2:**
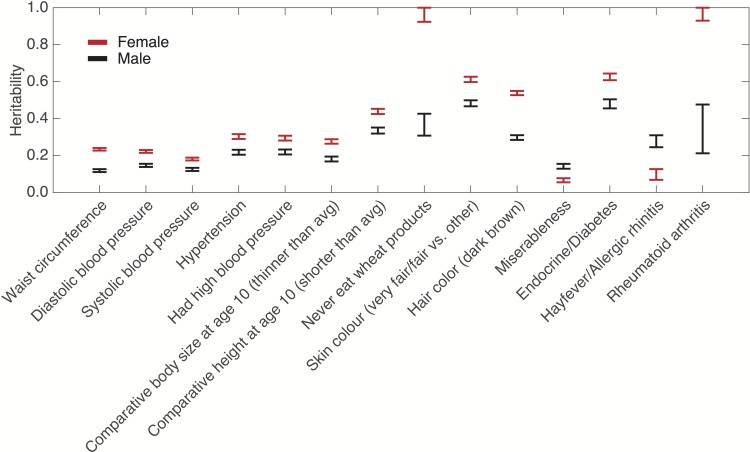
SNP heritability estimates and their standard errors of the traits in the UK Biobank that show significantly different SNP heritability in females and males after multiple testing correction. The heritability estimates of rheumatoid arthritis, endocrine/diabetes and wheat products intake reported here are based on genome-wide SNPs and will be markedly reduced when the major histocompatibility complex (MHC) region (chr6:25-35Mb) is excluded from analysis, and thus need to be interpreted with caution.

Heritability analysis stratified by sex identified a number of traits whose heritability showed significant difference in males and females after multiple testing correction (Fig 2). For example, the analyses of diastolic and systolic blood pressure, and self-reported hypertension and high blood pressure provided consistent evidence that the heritability of blood pressure related traits and diseases is significantly higher in females than in males.

**Fig 3:**
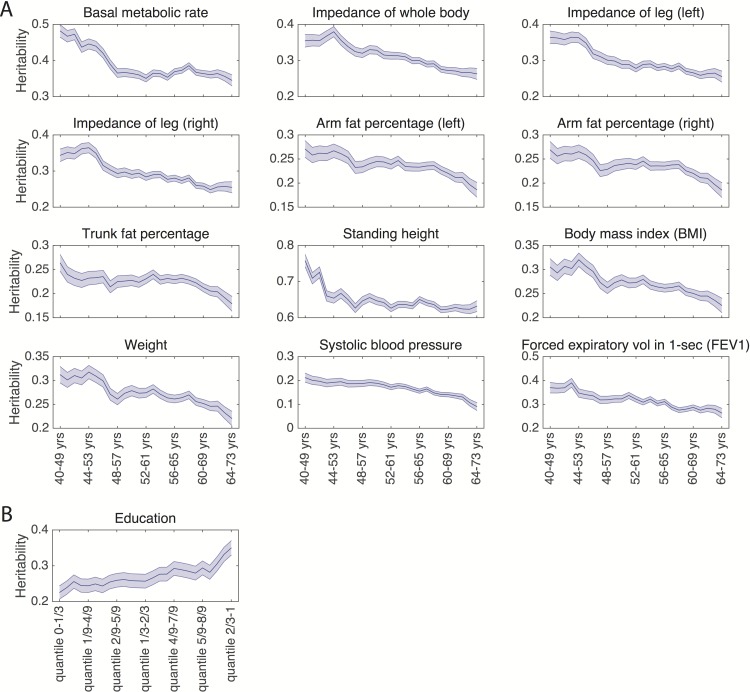
(A) The age-varying SNP heritability estimates and their standard errors (shaded region) for the 12 traits whose heritability significantly decreases with age after accounting for multiple testing correction; (B) The stratified heritability estimates and standard errors (shaded region) of education (has college or university degree or not), on which the socioeconomic status (SES) measured by the Townsend deprivation index has a significant moderating effect after multiple testing correction.

A majority of physical measures showed decreasing heritability with age (S3 Table). More specifically, 33 out of 50 physical measures had a significant decreasing trend in heritability estimates after accounting for multiple testing correction (mean slope of the 33 traits -0.0035, i.e., heritability estimates decrease by 3.5 percent per decade). The age-varying SNP heritability estimates and their standard errors for 12 traits that showed both significant slopes and significantly different heritability estimates between the first (40–49 years) and last age range (64–73 years) are shown in Fig 3A. S2 Fig shows the mean and standard deviation of the 12 traits in each age range.

When we stratified heritability by the Townsend deprivation index, a proxy for SES, education (has college or university degree or not) was the only trait on which SES had a significant moderating effect after accounting for multiple testing correction. Fig 3B shows that the heritability of education increases with increasing SES.

## Discussion

Estimating the heritability of complex, polygenic traits is an important component of defining the genetic basis of human phenotypes. In addition, heritability estimates provide a theoretical upper bound for the utility of genetic risk prediction models [9]. We calculated the common-variant heritability of 551 phenotypes derived from the interim data release of the UK Biobank, and confirmed that common genetic variation contributes to a broad array of quantitative traits and human diseases in the UK population. Two aspects of our work are particularly notable. First, we developed a computationally and memory efficient method that enabled us to calculate the most extensive population-based survey of SNP heritability to date. Second, we found that the heritability for a number of phenotypes is moderated by major demographic variables, demonstrating the dependence of heritability on population characteristics. We discuss each of these advances and the limitations of the biobank data and our analyses below.

Classical methods to estimate SNP heritability, such as GCTA (also known as the GREML method), rely on the restricted maximum likelihood (ReML) algorithm [3–5], which can give unbiased heritability estimates in quantitative trait analysis and non-ascertained case-control studies, and is statistically efficient when the trait is Gaussian distributed [10]. However, ReML is an iterative optimization algorithm, which is computationally and memory intensive, and thus can be difficult to apply when analyzing data sets with hundreds of thousands of subjects. An alternative and widely used SNP heritability estimation method is LD score regression, which is based on GWAS summary statistics and an external reference panel for the LD structure [8]. The approach can thus be easily applied to complex traits on which large-scale GWAS results are available, and allows meta-analysis of heritability estimates from different studies. Recently, LD score regression has been extended to partition heritability by functional annotation [11], and to estimate the genetic correlation between two traits [12,13]. However, when applying LD score regression to novel phenotypes in a large cohort, conducting GWAS is often time-consuming.

In the present study, we implemented a computationally and memory efficient moment-matching method for heritability estimation, which is closely related to the Haseman-Elston regression [14–16] and phenotype-correlation genetic-correlation (PCGC) regression [10], and produces unbiased SNP heritability estimates for both continuous and binary traits. The moment-matching method is theoretically less statistically efficient than the ReML algorithm (i.e., produces larger standard error on the point estimate) when analyzing quantitative traits, but the power loss is expected to be small [17] and is less of an issue given large sample sizes, such as in the UK Biobank. The moment-matching method is also mathematically equivalent to LD score regression if the following conditions are satisfied: (1) the out-of-sample LD scores estimated from the reference panel and the in-sample LD scores estimated from individual-level genotype data are identical; (2) the intercept in the LD score regression model is constrained to 1 (i.e., assuming that there is no confound and population stratification in the data); and (3) a particular weight is used in the LD score regression (more specifically, the reciprocal of the LD score, which is close to the default setting in the LD score regression software) [18]. Here, since we have constrained our analysis to a white British (Caucasian) sample and have accounted for potential population stratification by including top PCs of the genotype data as covariates, the two methods should produce similar estimates. See Box 1 for an empirical comparison between the moment-matching method, LD score regression and GCTA.

**Box 1**. An empirical comparison between the moment-matching method, LD score regression and genome-wide complex trait analysis (GCTA). To confirm our theoretical expectation of how the moment-matching method compares to LD score regression and GCTA, the most widely used SNP heritability estimation methods, we performed the following empirical analysis. For each trait in S1 Table, we randomly sampled 10,000 subjects and compared SNP heritability estimates from the moment-matching method, LD score regression (with constrained intercept and the regression weight set to the reciprocal of the LD score), and GCTA. S3 Fig shows that the moment-matching method produced virtually identical estimates to LD score regression, and consistent heritability and standard errors estimates with GCTA. The power loss of the moment-matching method relative to GCTA is negligible. Although the three methods produced consistent heritability estimates, the moment-matching method has much lower computational demand than the ReML algorithm and LD score regression. Specifically, using a single core of the (dual CPU) Intel Xeon 5472 3.0GHz processor and 7 Gb of virtual memory, GCTA takes approximately half an hour to analyze one trait (assuming that the genetic similarity matrix has been pre-computed) and each GWAS takes several hours, while the moment-matching method can produce the SNP heritability estimate of a trait in less than half a minute. When analyzing the full sample (~100,000 subjects), GCTA becomes very difficult to apply because its memory demand increases quadratically and computational complexity scales cubically with the sample size. It is also challenging to conduct stratification analyses across the phenotypic spectrum using LD score regression due to the large number of GWAS to be performed. In contrast, the moment-matching method only takes approximately 20 mins to complete the main heritability analysis and all the stratification analyses for a trait with 100,000 subjects using one CPU processor and 7Gb of memory. To summarize, we have used a method that balances statistical efficiency and computational burden with the flexibility to achieve our main goal in this study.

Using the moment-matching method, we found that a large number of traits we examined display significant heritability. For traits whose heritability has been intensively studied, our estimates are generally in line with prior studies. For example, twin and pedigree studies have estimated the heritability of human height and body mass index (BMI) to be approximately 80% and 40–60% [see e.g., 19–21], respectively, although recent studies have shown that heritability may be overestimated in family studies due to, for instance, improper modeling of common environment, assortative mating in humans, genetic interactions, and suboptimal statistical methods [10,22–25]. Using genome-wide SNP data from unrelated individuals, it has been shown that common SNPs explain a large proportion of the height and BMI variation in the population, although SNP heritability estimates are lower than twin estimates [4,5,26]. Specifically, the first GCTA analysis estimated the SNP heritability of human height to be 0.45 using relatively sparse genotyping data (approximately 300,000 SNPs) and showed that the estimate could be higher if imperfect LD between SNPs and causal variants are corrected [4]. A more recent study leveraging whole-genome sequencing data and imputed genetic variants concluded that narrow-sense heritability is likely to be 60–70% for height and 30–40% for BMI [27]. Here, we estimated the SNP heritability of human height and BMI to be 0.685+/-0.004 and 0.274+/-0.004, respectively, which are comparable to the expected range. The SNP heritability estimates of other complex traits of interest, such as age at menarche in girls (0.239+/-0.007), diastolic (0.184+/-0.004) and systolic (0.156+/-0.004) blood pressures, education (has colleague or university degree or not, 0.294+/-0.007), neuroticism (0.130+/-0.005), smoking (ever smoked or not, 0.174+/-0.006), asthma (0.340+/-0.010) and hypertension (0.263+/-0.007) were also more modest and lower than twin estimates, as expected [2].

Heritability is, by definition, a ratio of variances, reflecting the proportion of phenotypic variance attributable to individual differences in genotypes. Because the genetic architecture and non-genetic influences on a trait may differ depending on the population sampled, heritability itself may vary. Examples of this have been reported in the twin literature. In one well-known study, Turkheimer and colleagues [6] reported that the heritability of IQ is moderated by SES in a sample of 320 7-year-old twin pairs of mixed ancestry. In that study, the heritability of IQ was essentially 0 at the lowest end of SES but substantial at the highest end. Subsequent studies of the moderating effects of SES on the heritability of cognitive ability and development using twin designs have produced mixed results [28–33]. In our analysis, using SNP data, we observed no moderating effect of SES (as measured by the Townsend deprivation index) on the heritability of cognitive traits (including fluid intelligence), possibly due to the age range of participants in the UK Biobank (middle and old age) in contrast to many previous studies targeting childhood or early adulthood, and the cross-national differences in gene-by-SES interaction on intelligence as shown by a recent meta-analysis [34]. In addition, the brief cognitive tests available in the UK Biobank may have had limited sensitivity for capturing individual differences in IQ (see discussion below). On the other hand, the heritability of education showed significant interactions with SES, with increasing heritability at higher SES levels. Prior evidence has suggested that education has substantial genetic correlation with IQ and may be a suitable proxy phenotype for genetic analyses of cognitive performance [35]; thus our results may indirectly support earlier studies of the SES moderation of IQ heritability.

With two exceptions, significant sex differences we observed indicated greater heritability for women compared to men. Our results are consistent with findings from some twin studies but not others. For example, we found that women exhibited significantly greater heritability for measured waist circumference and blood pressure. Twin studies have also reported greater female heritability for waist circumference [36] but no substantial sex difference in heritability of blood pressure [2,37]. A substantial difference between the heritability of rheumatoid arthritis (RA) in males compared to females was observed, although the MHC region has a large impact on the SNP heritability estimates of autoimmune diseases, and thus this finding needs to be interpreted with caution (see discussion below). While RA is known to be more common in women, a twin analysis found no sex difference in heritability among Finnish and UK twin pairs, though power was limited in that analysis [38]. Intriguingly, greater heritability was observed among men for the personality trait of miserableness, a component of neuroticism, suggesting that environmental factors may be more influential for this trait among women or that measurement error differs by sex.

We examined age effects on heritability for a subset of variables and found that a number of physical measures indexing body size, adiposity, height, as well as systolic blood pressure and lung function, showed declining heritability with age. Age-related declines in heritability may reflect the cumulative effect of environmental perturbations over the lifespan. Prior twin studies of age effects on the heritability of anthropometric traits in adults have had inconsistent results [39–41]. Haworth and colleagues showed that the heritability of BMI increases over childhood [42]. A recent meta-analysis of 32 twin studies documented a non-monotonic relationship between BMI heritability and age (from childhood to late adulthood), with a peak around age 20 and decline thereafter [43]. An age-related decline in indices of body size may reflect a decreasing contribution of genetically-regulated growth processes over the lifespan. However, we were unable to assess the entire trajectory of heritability due to the age range (40–73 years) of the UK Biobank participants. Some but not all studies have also suggested varying or declining heritability with age for blood pressure, lung function and age at first birth [39,44–50].

Our results should be interpreted in light of the limitations associated with the biobank data. First, the UK Biobank is restricted to middle and old age groups, which may be subject to sample selection bias. For example, older and physically/cognitively impaired subjects may be underrepresented in the study, which may have an impact on the heritability estimates stratified by age. Mortality selection can also alter the results of genetic analyses as shown by recent analyses [51]. In addition, the UK Biobank participants comprised a relatively high proportion of well-educated, skilled professionals [52], potentially leading to the underrepresentation or restricted range of certain traits such as smoking relative to other cohorts. Therefore, our heritability estimates may be specific to this UK population and may not generalize to other settings or ancestry groups. Second, although the UK Biobank has collected a wealth of phenotypes, measurements associated with a particular phenotypic domain may not be comprehensive. For example, only five cognitive tests were included in the UK Biobank. The reasoning task (fluid intelligence test) was brief and had a narrow range; the reaction time was averaged from a small number of trials; and the visual memory test (pairs-matching test) had a significant floor effect (a large number of participants made zero or very few mistakes, and thus the scores do not fully reflect individual differences). In addition, all cognitive tests had relatively low reliability across repeat measurements [53]. These noisy measurements may thus downwardly bias heritability estimates of cognition. The Townsend deprivation index, which we used to stratify phenotypes, was calculated based on the national census output area of each participant in which their postcode was located at the time of recruitment, and thus can only serve as a proxy for SES. Third, the phenotypes were limited to those for which we had sufficient data to estimate heritability with adequate precision. Therefore, diseases with low prevalence in the sampled population were not well represented in our analysis. We expect to analyze traits with lower prevalence (e.g., 0.5%) when the genetic data for all UK Biobank participants becomes available. We also assumed in our analysis that the population prevalence of a binary trait is identical to the observed sample prevalence, but diseases such as schizophrenia and stroke are naturally under-ascertained and thus their sample prevalence is often lower than population prevalence. In addition, we note that since we used medical history to define cases and controls, the prevalence of many diseases we investigated reflected lifetime prevalence, which may be different from cross-sectional prevalence used in other studies. We also binarized categorical (multinomial or ordinal) variables to facilitate analysis, but this might not optimally represent variation in these variables with respect to heritability. Fourth, a substantial fraction of the phenotypes we examined were based on self-report or diagnostic (ICD-10) codes, which may or may not validly capture the phenotypes they represent. For example, a recent UK Biobank study shows that 51% of the participants who reported RA were not on RA-relevant medication, a proxy measure of valid diagnosis [54]. However, our head-to-head comparison of the heritability estimates between self-reported illness and ICD-10 codes showed largely consistent results, indicating that both phenotypic approaches at least captured comparable variations in these phenotypes. Prior research evaluating phenotypes derived from electronic health records (EHR) indicate that greater phenotypic validity can be achieved when diagnostic codes are supplemented with text mining methods [55–58]. The specificity of the disease codes might also be improved by leveraging the medication records in the UK Biobank.

Methodologically, our SNP heritability estimation approach, despite its superior computational and memory performance compared to existing methods, also has several limitations. First, heritability estimation always relies on a number of assumptions on the genetic architecture. For example, the moment-matching method we used here, as well as the established GCTA and LD score regression approaches, implicitly assumes that the causal SNPs are randomly spread over the genome, which is independent of the MAF spectrum and the LD structure, and the effect sizes of causal SNPs are Gaussian distributed and have a specific relationship to their MAFs. Although it has been shown that SNP heritability estimates are reasonably robust to many of these modeling assumptions [59], the estimates can be biased if, for instance, causal SNPs are rarer or more common than uniformly distributed on the MAF spectrum, or are enriched in high or low LD regions across the genome. For example, the heritability estimates for some autoimmune diseases such as psoriasis and RA dropped dramatically when the MHC region (chr6:25-35Mb) was removed when constructing the genetic similarity matrix, indicating, as expected, that causal variants for these diseases are disproportionally enriched in the MHC region. S4 Table lists all the traits whose heritability estimates decreased by 0.2 or more when the MHC region was taken out, and thus need to be interpreted with caution. Methods to correct for MAF properties and region-specific LD heterogeneity of causal variants have been proposed [27,59,60]. For example, we can stratify MAF and LD structure into different bins, compute a genetic similarity matrix within each bin, and fit a mixed effects model with multiple variance components [27,60]. This approach can give heritability estimates that are more robust to properties of the underlying genetic architecture, but has the downside of increased computational burden and reduced statistical power. A different direction to explore is to estimate SNP heritability using imputed data (in contrast to the genotype data here), which might capture more genetic variation from rare variants, or common variants that are not well tagged by the genotyped SNPs, and thus lead to increased heritability estimates. Second, heritability analysis models, including the one we employed in the present study, typically assume that genetic and environmental effects are independent, i.e., no gene-by-environment (GxE) interaction exists. This is certainly a simplification of the real world where GxE interactions are expected for many complex traits. Recent computational studies have also shown that ignoring GxE interactions in heritability analysis can produce biased estimates [25]. However, modeling GxE would require collecting relevant environmental variables for each phenotype and more sophisticated statistical modeling approaches, e.g., incorporating multiple random effects in the heritability analysis model [5,61]. Due to the limited measurements of environment collected by the UK Biobank, and the extensive analyses we have conducted across the phenotypic spectrum, explicitly modeling the environmental factors and GxE interactions is not feasible. We therefore took an alternative approach to examine the moderating effects of three major demographic variables on heritability estimates by stratifying samples. Of note, consistent heritability estimates across different levels of the stratifying variable do not completely eliminate the potential existence of GxE interactions. Specifically, recent studies have identified genetic heterogeneity in human traits such as BMI and fertility [62,63], indicating that the genetic architecture of a trait may be different across environments (i.e., the genetic correlation of a trait in different environments may be significantly smaller than 1) even if the overall heritability estimates are similar. Dissection of common and unique environmental influences and their interactive effects with genetics on different complex traits, and the shared and unique genetic effects across environments are important future directions to explore. Lastly, as reviewed in [64], a number of empirical genetic similarity measurements computable from genome-wide SNP data have been proposed, which, when utilized in heritability analysis, can give different estimates with different interpretations. In addition, recent studies have argued that estimation error associated with genetic similarity measurements and the ill-posedness of the empirical genetic similarity matrix may produce unstable and unreliable SNP heritability estimates [65]. However, this is an area under active investigation and debate [64–67]. Here, as the first study to screen all UK Biobank variables and provide an overview of the distribution of SNP heritability across different trait domains, and to examine the effect of potential modifying variables on heritability estimates, we used a straightforward and classical modeling approach that is most widely used. To obtain more insights into the genetic architecture and find the most appropriate and robust model for each individual trait, more systematic investigation is needed.

In sum, using a computationally and memory efficient approach, we provide estimates of the SNP heritability for 551 complex traits across the phenome captured in the population-based UK Biobank. We further identify phenotypes for which the contribution of genetic variation is modified by demographic factors. These results underscore the importance of considering population characteristics in interpreting heritability, highlight phenotypes and subgroups that may warrant priority for genetic association studies, and may inform efforts to apply genetic risk prediction models for a broad range of human phenotypes.

## Materials and methods

### Ethics statement

This study utilized deidentified data from the baseline assessment of the UK Biobank, a prospective cohort study of 500,000 individuals (age 40–69 years) recruited across Great Britain during 2006–2010 [7]. The protocol and consent were approved by the UK Biobank’s Research Ethics Committee.

### Participants and data sources

The UK Biobank collected phenotypic data from a variety of sources including questionnaires regarding mental and physical health, food intake, family history and lifestyle, a baseline physical assessment, computerized cognitive testing, linkage with health records, and blood samples for biochemical and DNA analysis. Details about the UK Biobank project are provided at http://www.ukbiobank.ac.uk. Data for the current analyses were obtained under an approved data request (Ref: 13905).

### Genotyping and quality control

The interim release of the genotype data for the UK Biobank (downloaded on Mar 3, 2016) comprises 152,736 samples. Two closely related arrays from Affymetrix, the UK BiLEVE Axiom array and the UK Biobank Axiom array, were used to genotype approximately 800,000 markers with good genome-wide coverage. Details of the design of the arrays and sample processing can be found at http://biobank.ctsu.ox.ac.uk/crystal/refer.cgi?id=146640 and http://biobank.ctsu.ox.ac.uk/crystal/refer.cgi?id=155583.

Prior to the release of the genotype data, stringent quality control (QC) was performed at the Wellcome Trust Centre for Human Genetics, Oxford, UK. Procedures were documented in detail at http://biobank.ctsu.ox.ac.uk/crystal/refer.cgi?id=155580. We leveraged the QC metrics made available by the UK Biobank and removed samples that had mismatch between genetically inferred sex and self-reported sex, samples that had high genotype missingness or extreme heterozygosity not explained by mixed ancestry or increased levels of marriage between close relatives, and one individual from each pair of the samples that were 3^rd^ degree or more closely related relatives. We restricted our analysis to subjects that were self-reported white British and confirmed by principal component analysis (PCA) to be Caucasians. We further filtered out genetic markers that had high missing rate (>1%), low minor allele frequency (<1%), significant deviation from Hardy-Weinberg equilibrium (*p*<1e-7), and subjects that had high missing genotype rate (>1%). 108,158 subjects (age 40–73 years; female 52.84%) and 486,175 SNPs remained for analysis after QC. S4 Fig shows the age distribution of the subjects that passed QC. The genetic similarity matrix was computed using all genotyped autosomal SNPs. All genetic analyses were performed using PLINK 1.9 (https://www.cog-genomics.org/plink2) [68].

### Phenotypic variables

We analyzed every trait available to us that had a sufficient sample size to produce a heritability estimate with its standard error smaller than 0.1. The traits can be classified into the following 11 domains as defined by the UK Biobank: cognitive functions, early life factors, health and medical history, life style, physical measures, psychosocial factors, sex-specific factors and sociodemographics. For continuous traits, we excluded samples that were more than 5 standard deviations away from the population mean to avoid extreme outliers and data recording errors. We only analyzed binary traits that had prevalence greater than 1% in the sample, so that we had enough statistical power to get reliable heritability estimates. We typically binarized categorical variables at a meaningful threshold close to the median and then analyzed them as binary traits. For the specific cutoff-points used to binarize each categorical variable, see S1 Table.

We also analyzed a large number of self-reported illness codes and hospital in-patient diagnosis codes. Self-reported cancer and non-cancer illness codes were obtained through a verbal interview by a trained nurse at the UK Biobank assessment center on past and current medical conditions. Hospital in-patient diagnoses were obtained through medical records and were coded according to the International Classification of Diseases version-10 (ICD-10). Disease codes for each domain (self-reported cancer, self-reported non-cancer illness, and ICD-10) were organized in a hierarchical tree structure; codes closer to the root of the tree are often less specific and have larger prevalence, while codes closer to the leaves are more specific but have lower prevalence. We analyzed every disease code that had prevalence greater than 1% in the sample. 15 ICD-10 codes were excluded due to small sample sizes and large standard errors (>0.1) on heritability estimates (N70-N77 Inflammatory diseases of female pelvic organs; O20-O29 Other maternal disorders predominantly related to pregnancy; O80-O84 Delivery; N50 Other disorders of male genital organs; N80 Endometriosis; N81.1 Cystocele; N81.2 Incomplete uterovaginal prolapse; N83 Noninflammatory disorders of ovary—Fallopian tube and broad ligament; N83.2 Other and unspecified ovarian cysts; N84.1 Polyp of cervix uteri; N92.1 Excessive and frequent menstruation with irregular cycle; N93 Other abnormal uterine and vaginal bleeding; O68 Labour and delivery complicated by foetal stress [distress]; O70.1 Second degree perineal laceration during delivery; O80 Single spontaneous delivery). We also employed a data-driven approach to determine if a disease is sex-specific. More specifically, if the sample prevalence of a disease in males was more than 100 times larger than the sample prevalence in females, we defined the disease as male-specific and the analysis was restricted to males. The same approach was used to find female-specific diseases. See S2 Table for all the disease codes we analyzed.

### Heritability estimation

We consider the linear random effect model ***y*** = ***g*** + ***e***, where an *N*-dimensional trait ***y*** is partitioned into the sum of additive genetic effects ***g*** and unique (subject-specific) environmental effects ***e***. The covariance structure of ***y*** is $cov[y]=\sigma_{g}^{2}K+\sigma_{e}^{2}I$, where ***K*** is the empirical genetic similarity matrix for each pair of individuals estimated from genome-wide SNP data [4,5], ***I*** is an identity matrix, $\sigma_{g}^{2}$ and $\sigma_{e}^{2}$ are the total additive genetic variance captured by genotyped common SNPs and the variance of unique environmental factors across individuals, respectively. SNP heritability is then defined as $h_{SNP}^{2}=\sigma_{g}^{2}/(\sigma_{g}^{2}+\sigma_{e}^{2})=\sigma_{g}^{2}/\sigma_{p}^{2}$, which measures the total phenotypic variance $\sigma_{p}^{2}$ that can be explained by total additive genetic variance tagged by genotyped SNPs, and is a lower bound for the narrow-sense heritability *h*^2^. When covariates need to be incorporated into the model, i.e., ***y*** = ***Xβ*** + ***g*** + ***e***, where ***X*** is an *N* × *q* covariate matrix and ***β*** is a vector of fixed effects, an *N* × (*N* − *q*) matrix ***U*** always exists, which satisfies ***U***^*T*^***U*** = ***I***, ***UU***^*T*^ = ***P***_**0**_, ***U***^*T*^***X*** = **0**, and ***P***_**0**_ = ***I*** − ***X***(***X***^*T*^***X***)^−1^***X***^*T*^. Applying ***U***^*T*^ to both sides of the model removes the covariate matrix [69,70].

To obtain unbiased estimates of $\sigma_{g}^{2}$ and $\sigma_{e}^{2}$, we used a computationally efficient moment-matching approach [70,71], which is closely related to the Haseman-Elston regression [14–16] and phenotype-correlation genetic-correlation (PCGC) regression [10], and mathematically equivalent to the LD score regression under certain conditions [8,18]. Specifically, we regress the empirical estimate of the phenotypic covariance onto the matrices ***K*** and ***I***: $vec[yy^{T}]=\sigma_{g}^{2}vec[K]{+\sigma }_{e}^{2}vec[I]+\epsilon$, where vec[⋅] is the matrix vectorization operator that converts a matrix into a vector by stacking its columns, and ***ϵ*** is the residual of the regression. The ordinary least squares (OLS) estimator of this multiple regression problem can be explicitly written as ${\hat{\sigma }}_{g}^{2}=\frac{1}{v_{K}}y^{T}(K-\tau I)y$ and ${\hat{\sigma }}_{e}^{2}=\frac{1}{v_{K}}y^{T}(\kappa I-\tau K)y$, where *τ* = tr[***K***]/*N*, *κ* = tr[***K***^**2**^]/*N*, and *v*_*K*_ = *N*(*κ* − *τ*^2^). SNP heritability is then estimated as ${\hat{h}}_{SNP}^{2}={\hat{\sigma }}_{g}^{2}/({\hat{\sigma }}_{g}^{2}+{\hat{\sigma }}_{e}^{2})={\hat{\sigma }}_{g}^{2}/{\hat{\sigma }}_{p}^{2}$.

To estimate the sampling variance of ${\hat{h}}_{SNP}^{2}$, we follow Visscher et al. [17] and make two assumptions: (1) the off-diagonal elements in the empirical genetic similarity matrix ***K*** are small, such that ***K*** ≈ ***I*** and $V=cov[y]=\sigma_{g}^{2}K+\sigma_{e}^{2}I\approx \sigma_{p}^{2}I$; and (2) the phenotypic variance $\sigma_{p}^{2}$ can be estimated with very high precision. We thus have $var[{\hat{\sigma }}_{g}^{2}]=\frac{2}{v_{K}^{2}}tr[(K-\tau I)V(K-\tau I)V]\approx {2\sigma }_{p}^{4}/v_{K}$, and $var[{\hat{h}}_{SNP}^{2}]\approx 2/v_{K}$. This estimator coincides with existing results in the literature [17]. We note that the calculation of the variance of ${\hat{\sigma }}_{g}^{2}$ relies on an additional assumption that the trait ***y*** is Gaussian distributed and thus may be suboptimal for binary traits. However, Visscher and colleagues have empirically shown that this sampling variance approximation is accurate for both continuous and binary traits when the sample size is large [17] (also see S3 Fig).

We note that for large sample size *N*, the *N* × *N* genetic similarity matrix ***K*** and residual forming matrix ***P***_**0**_ can be very large, making the computation memory intensive. We have developed a memory efficient algorithm that can iteratively load columns (or block columns) of ***K*** into the memory to compute the SNP heritability estimate, and does not need to explicitly compute ***P***_**0**_ or any other *N* × *N* matrices. See S1 Text for details. Matlab and Python implementations of the algorithm are available at https://github.com/chiayenchen/mmhe.

For binary traits, the above calculation gives a heritability estimate on the observed scale, which is dependent on prevalence of the trait in the population. We transformed this heritability estimate to the underlying liability scale under the assumption of a classical liability threshold model [72,73], which makes heritability estimates independent of prevalence and thus comparable across traits. Specifically, heritability estimate on the liability scale can be obtained using a linear transformation of the heritability on the observed scale: ${\hat{h}}_{SNP,L}^{2}={c\hat{h}}_{SNP}^{2}$, where *c* = *P*(1 − *P*)/*φ*(*t*)^2^, *P* is the population prevalence, *t* = Φ^−1^(1 − *P*) is the liability threshold, Φ is the cumulative distribution function of the standard normal distribution, and *φ* is the density function of the standard normal distribution [3,74]. Since the UK Biobank is not designed to be ascertained for particular diseases, we assumed that population prevalence is identical to sample prevalence. The sampling variance of the heritability estimate can be transformed accordingly: $var[{\hat{h}}_{SNP,L}^{2}]=c^{2}var[{\hat{h}}_{SNP}^{2}]$.

### Statistical analysis

In all heritability analyses, we included genotyping array, UK Biobank assessment center, age at recruitment and top 10 principal components (PCs) of the genotype data as covariates. Other covariates such as sex and handedness (e.g., when analyzing the grip strength of the left/right hand) were adjusted where appropriate. See S1 Table for the set of covariates we included in the model when estimating the heritability for each trait. To compute PCs of the genotype data, we performed pairwise linkage disequilibrium (LD) based SNP pruning at *R*^2^>0.02 and excluded SNPs in the major histocompatibility complex (MHC) region (chr6:25-35Mb) and chromosome 8 inversion (chr8:7-13Mb). Top PCs were then computed using flashPCA [75] on the pruned data, which employs an efficient randomized algorithm and is thus scalable to large data sets with hundreds of thousands of individuals.

To examine how heritability estimates are modified by sex, we estimated heritability for each non-sex-specific trait in males and females separately. For binary traits, sample prevalence was calculated in each sex. To test if heritability estimates are significantly different by sex, we assumed that the two SNP heritability estimates to be contrasted, ${\hat{h}}_{A}$ and ${\hat{h}}_{B}$, are independent and approximately Gaussian distributed, and computed the *z*-score of their difference: $z=({\hat{h}}_{A}-{\hat{h}}_{B})/\sqrt{{\hat{se}}_{A}^{2}+{\hat{se}}_{B}^{2}}$, where ${\hat{se}}_{A}^{2}$ and ${\hat{se}}_{B}^{2}$ are standard error estimates of ${\hat{h}}_{A}$ and ${\hat{h}}_{B}$, respectively. A *p*-value can then be computed as *p* = 2 ∙ Φ(−|*z*|), where Φ is the cumulative distribution function of the standard normal distribution.

To examine whether SNP heritability estimates vary with age, we used a sliding window approach and estimated heritability for every age range of 10 years (i.e., 40–49 years, 41–50 years, …, 64–73 years) by stratifying the samples. For binary traits, sample prevalence was calculated in each age range separately. We assessed whether heritability estimates exhibited a linear trend with age by fitting a regression model, ${\hat{h}}_{k}^{2}=\alpha +{\bar{age}}_{k}\gamma +\epsilon_{k}$, where ${\hat{h}}_{k}^{2}$ is the heritability estimate in the *k*-th age range, ${\bar{age}}_{k}$ is the mean of the age range, *α* is an intercept, *γ* is the slope and *ϵ*_*k*_ is the residual of the regression, and testing whether *γ* is significantly different from zero. We weighted heritability estimates by the inverse of their standard errors when fitting the regression model, and thus put more emphasis on estimates with better precision. We only analyzed physical and cognitive measures, and did not consider disease codes and medical history in age stratification analyses because age at recruitment does not reflect disease onset.

Similarly, we used a sliding window approach to estimate the SNP heritability for each trait from the bottom 1/3 quantile to the top 1/3 quantile of the Townsend deprivation index at recruitment, a measure of material deprivation within the population of a given area. For binary traits, sample prevalence was calculated in each SES bin separately. For traits that do not reflect the status of participants at the time of recruitment (e.g., medical history and early-life factors), we have implicitly made an assumption that the SES of participants had not changed dramatically throughout their lives.

To account for multiple testing in our stratification analyses, we corrected the *p*-values using the effective number of independent traits we analyzed. Specifically, for each stratification analysis (sex, age and SES), we calculated the Pearson correlation coefficient for each pair of the traits using their overlapping samples. The correlation between traits that had no sample overlap, e.g., male- and female-specific factors, was set to zero. We then conducted a principal component analysis (PCA) to the constructed phenotypic correlation matrix, and estimated the effective numbers of independent traits that explained 99% of the total phenotypic variation in sex, age and SES stratification analyses to be 400, 31 and 440, respectively. Finally, we multiplied uncorrected *p*-values by the corresponding effective number of independent traits to obtain corrected *p*-values.

## Supporting information

S1 Text The moment-matching method for SNP heritability analysis. (DOCX)

S1 Table The SNP heritability estimates, standard error estimates, sample sizes, covariates adjusted, prevalence in the sample (for binary traits) and other relevant information for all non-disease traits analyzed in the UK Biobank. SNP Heritability estimates stratified by sex and socioeconomic status (SES) measured by the Townsend deprivation index are also presented.
(XLSX)

S2 Table The SNP heritability estimates, standard error estimates, sample sizes, prevalence in the sample and other relevant information for all self-reported illness codes and ICD-10 codes analyzed in the UK Biobank. SNP Heritability estimates stratified by sex and socioeconomic status (SES) measured by the Townsend deprivation index are also presented.
(XLSX)

S3 Table SNP Heritability estimates stratified by age. (XLSX)

S4 Table Traits whose SNP heritability estimates decrease by 0.2 or more when the major histocompatibility complex (MHC) region (chr6:25-35Mb) is removed when computing the genetic similarity matrix. (XLSX)

S1 Fig (A) A breakdown of the 79 self-reported non-cancer illness codes into different functional domains; (B) A breakdown of the 194 ICD-10 codes into different functional domains.
(TIFF)

S2 Fig The mean and standard deviation (shaded region) of the 12 traits whose SNP heritability estimates significantly decrease with age. (TIFF)

S3 Fig A comparison of the SNP heritability and standard error (SE) estimates from the moment-matching method, genome-wide complex trait analysis (GCTA) and LD score regression (with constrained intercept and the regression weight set to the reciprocal of the LD score), by randomly sampling 10,000 subjects for each trait in S1 Table. (TIFF)

S4 Fig Age distribution of the sample analyzed in the UK Biobank. (TIFF)
