# Correction: Phenome-wide heritability analysis of the UK Biobank

**DOI:** 10.1371/journal.pgen.1007228

**Published:** 2018-02-09

**Authors:** Tian Ge, Chia-Yen Chen, Benjamin M. Neale, Mert R. Sabuncu, Jordan W. Smoller

There is an error in the final sentence of the Results. The correct sentence is: Fig 3B shows that the heritability of education increases with increasing deprivation (ie decreasing SES).

There are errors in the final two sentences of the fifth paragraph of the Discussion. The correct sentences are: On the other hand, the heritability of education showed significant interactions with SES, with increasing heritability at lower SES levels. Prior evidence has suggested that education has substantial genetic correlation with IQ and may be a suitable proxy phenotype for genetic analyses of cognitive performance [35]; thus our results may indirectly support earlier studies of the SES moderation of IQ heritability, though in the opposite direction of that reported in twin study data.
