# Supplementary material for: Phenome-wide heritability analysis of the UK Biobank
Source: PLoS Genet. 2017 Apr 7;13(4):e1006711. doi: 10.1371/journal.pgen.1006711 (PMC5400281; doi:10.1371/journal.pgen.1006711)
Supplement: S1 Text — (DOCX) [file pgen.1006711.s001.docx]

**Supplementary Information for “Phenome-wide Heritability Analysis of the UK Biobank”**

Tian Ge^1,2,3^, Chia-Yen Chen^2,3,4^, Benjamin M. Neale^2,3,4^, Mert R. Sabuncu^1,5,*^, and Jordan W. Smoller^2,3,*^

^1^Athinoula A. Martinos Center for Biomedical Imaging, Massachusetts General Hospital / Harvard Medical School, Charlestown, MA 02129, USA;

^2^Psychiatric and Neurodevelopmental Genetics Unit, Center for Genomic Medicine, Massachusetts General Hospital, Boston, MA 02114, USA;

^3^Stanley Center for Psychiatric Research, Broad Institute of MIT and Harvard, Cambridge, MA 02138, USA;

^4^Analytic and Translational Genetics Unit, Center for Genomic Medicine, Massachusetts General Hospital, Boston, MA 02114, USA;

^5^School of Electrical and Computer Engineering and School of Biomedical Engineering, Cornell University, Ithaca, NY 14853, USA.

*MRS and JWS contributed equally to this work.

Correspondence to: [tge1@mgh.harvard.edu](mailto:tge1@mgh.harvard.edu) (TG) or [jsmoller@hms.harvard.edu](mailto:jsmoller@hms.harvard.edu) (JWS)

**The Moment-matching Method for SNP Heritability Estimation**

We consider the linear random effect model $\boldsymbol{y}=\boldsymbol{g}+\boldsymbol{e}$, where an $N$-dimensional trait $\boldsymbol{y}$ is partitioned into the sum of additive genetic effects $\boldsymbol{g}$ and unique (subject-specific) environmental effects $\boldsymbol{e}$. The covariance structure of $\boldsymbol{y}$ is $\mathrm{cov}\left[ \boldsymbol{y} \right]=\sigma_{g}^{2}\boldsymbol{K}+\sigma_{e}^{2}\boldsymbol{I}_{N}$, where $\boldsymbol{K}$ is the empirical genetic similarity matrix for each pair of individuals estimated from genome-wide SNP data, $\boldsymbol{I}_{N}$ is an $N\times N$ identity matrix, $\sigma_{g}^{2}$ and $\sigma_{e}^{2}$ are the total additive genetic variance captured by genotyped common SNPs and the variance of unique environmental factors across individuals, respectively.

To obtain unbiased estimates of $\sigma_{g}^{2}$ and $\sigma_{e}^{2}$, we regress the empirical estimate of the phenotypic covariance onto the matrices $\boldsymbol{K}$ and $\boldsymbol{I}$: $\mathrm{vec}\left[ \boldsymbol{y}\boldsymbol{y}^{T} \right]=\sigma_{g}^{2}\mathrm{vec}\left[ \boldsymbol{K} \right]{+\sigma}_{e}^{2}\mathrm{vec}\left[ \boldsymbol{I} \right]+\boldsymbol{\epsilon}$, where $\mathrm{vec}[\cdot]$ is the matrix vectorization operator that converts a matrix into a vector by stacking its columns, and $\boldsymbol{\epsilon}$ is the residual of the regression. The ordinary least squares (OLS) estimator of this multiple regression problem can be obtained by solving the linear system:

$$\left[ \begin{matrix} \mathrm{tr}\left[ \boldsymbol{K}^{2} \right] & \mathrm{tr}\left[ \boldsymbol{K} \right] \\ \mathrm{tr}\left[ \boldsymbol{K} \right] & N \end{matrix} \right]\left[ \begin{matrix} \sigma_{g}^{2} \\ \sigma_{e}^{2} \end{matrix} \right]\boldsymbol{=}\left[ \begin{matrix} \boldsymbol{y}^{T}\boldsymbol{Ky} \\ \boldsymbol{y}^{T}\boldsymbol{y} \end{matrix} \right]\boldsymbol{.}$$

In the presence of covariates, i.e., $\boldsymbol{y}=\boldsymbol{X\beta}+\boldsymbol{g}+\boldsymbol{e}$, where $\boldsymbol{X}$ is an $N\times q$ covariate matrix and $\boldsymbol{\beta}$ is a vector of fixed effects, an $N\times(N-q)$ matrix $\boldsymbol{U}$ always exists, which satisfies $\boldsymbol{U}^{T}\boldsymbol{U=I}$, $\boldsymbol{U}\boldsymbol{U}^{T}\boldsymbol{=}\boldsymbol{P}_{\boldsymbol{0}}$, $\boldsymbol{U}^{T}\boldsymbol{X=0}$, and $\boldsymbol{P}_{\boldsymbol{0}}\boldsymbol{=I-X}{\boldsymbol{(}\boldsymbol{X}^{T}\boldsymbol{X)}}^{-1}\boldsymbol{X}^{T}$. Applying $\boldsymbol{U}^{T}$ to both sides of the model removes the covariate matrix and gives $\boldsymbol{U}^{T}\boldsymbol{y}=\boldsymbol{U}^{T}\boldsymbol{g}+\boldsymbol{U}^{T}\boldsymbol{e}$. The covariance structure of the transformed trait is $\mathrm{cov}\left[ \boldsymbol{U}^{T}\boldsymbol{y} \right]=\sigma_{g}^{2}\boldsymbol{U}^{T}\boldsymbol{KU}+\sigma_{e}^{2}\boldsymbol{I}_{N-q}$, and the linear system becomes

$$\left[ \begin{matrix} \mathrm{tr}\left[ \boldsymbol{P}_{\boldsymbol{0}}\boldsymbol{K}\boldsymbol{P}_{\boldsymbol{0}}\boldsymbol{K} \right] & \mathrm{tr}\left[ \boldsymbol{P}_{\boldsymbol{0}}\boldsymbol{K} \right] \\ \mathrm{tr}\left[ \boldsymbol{P}_{\boldsymbol{0}}\boldsymbol{K} \right] & N-q \end{matrix} \right]\left[ \begin{matrix} \sigma_{g}^{2} \\ \sigma_{e}^{2} \end{matrix} \right]\boldsymbol{=}\left[ \begin{matrix} \boldsymbol{y}^{T}\boldsymbol{P}_{\boldsymbol{0}}\boldsymbol{K}\boldsymbol{P}_{\boldsymbol{0}}\boldsymbol{y} \\ \boldsymbol{y}^{T}\boldsymbol{P}_{\boldsymbol{0}}\boldsymbol{y} \end{matrix} \right]\boldsymbol{.}$$

We note that for large sample size $N$, the $N\times N$ genetic similarity matrix $\boldsymbol{K}$ and the $N\times N$ residual forming matrix $\boldsymbol{P}_{\boldsymbol{0}}$ can be very large, making the computation of $\boldsymbol{y}^{T}\boldsymbol{P}_{\boldsymbol{0}}\boldsymbol{y}$, $\boldsymbol{y}^{T}\boldsymbol{P}_{\boldsymbol{0}}\boldsymbol{K}\boldsymbol{P}_{\boldsymbol{0}}\boldsymbol{y}$, $\mathrm{tr}\left[ \boldsymbol{P}_{\boldsymbol{0}}\boldsymbol{K} \right]$, and $\mathrm{tr}\left[ \boldsymbol{P}_{\boldsymbol{0}}\boldsymbol{K}\boldsymbol{P}_{\boldsymbol{0}}\boldsymbol{K} \right]$ memory intensive. To reduce the memory demand, we note that (1) the number of covariates $q$ is typically orders of magnitude smaller than $N$; (2) the quantities $\boldsymbol{y}^{T}\boldsymbol{K}$, $\boldsymbol{X}^{T}\boldsymbol{K}$, $\mathrm{tr}\left[ \boldsymbol{K} \right]=\sum_{i} k_{ii}$, and $\mathrm{tr}\left[ \boldsymbol{K}^{2} \right]=\sum_{ij} k_{ij}^{2}$ can be computed by iteratively reading columns (or block columns) of $\boldsymbol{K}$ into the memory; and (3) we have the following derivations:

$$\boldsymbol{y}^{T}\boldsymbol{P}_{\boldsymbol{0}}\boldsymbol{y=}\boldsymbol{y}^{T}\boldsymbol{y-}\boldsymbol{y}^{T}\boldsymbol{X}\left( \boldsymbol{X}^{T}\boldsymbol{X} \right)^{-1}\boldsymbol{X}^{T}\boldsymbol{y},$$

$$\mathrm{tr}\left[ \boldsymbol{P}_{\boldsymbol{0}}\boldsymbol{K} \right]=\mathrm{tr}\left[ \boldsymbol{K} \right]-\mathrm{tr}\left[ \boldsymbol{X}^{T}\boldsymbol{KX}\left( \boldsymbol{X}^{T}\boldsymbol{X} \right)^{-1} \right],$$

$$\boldsymbol{y}^{T}\boldsymbol{P}_{\boldsymbol{0}}\boldsymbol{K}\boldsymbol{P}_{\boldsymbol{0}}\boldsymbol{y=}\boldsymbol{y}^{T}\boldsymbol{Ky-}2\boldsymbol{y}^{T}\boldsymbol{X}\left( \boldsymbol{X}^{T}\boldsymbol{X} \right)^{-1}\boldsymbol{X}^{T}\boldsymbol{Ky+}\boldsymbol{y}^{T}\boldsymbol{X}\left( \boldsymbol{X}^{T}\boldsymbol{X} \right)^{-1}\boldsymbol{X}^{T}\boldsymbol{KX}\left( \boldsymbol{X}^{T}\boldsymbol{X} \right)^{-1}\boldsymbol{X}^{T}\boldsymbol{y},$$

$$\mathrm{tr}\left[ \boldsymbol{P}_{\boldsymbol{0}}\boldsymbol{K}\boldsymbol{P}_{\boldsymbol{0}}\boldsymbol{K} \right]=\mathrm{tr}\left[ \boldsymbol{K}^{2} \right]-2\mathrm{tr}\left[ \left( \boldsymbol{X}^{T}\boldsymbol{X} \right)^{-1}\boldsymbol{X}^{T}\boldsymbol{KKX} \right]+\mathrm{tr}\left[ \boldsymbol{X}\left( \boldsymbol{X}^{T}\boldsymbol{X} \right)^{-1}\boldsymbol{X}^{T}\boldsymbol{KX}\left( \boldsymbol{X}^{T}\boldsymbol{X} \right)^{-1}\boldsymbol{X}^{T}\boldsymbol{K} \right].$$

Therefore, it can be seen that once $\boldsymbol{y}^{T}\boldsymbol{K}$, $\boldsymbol{X}^{T}\boldsymbol{K}$, $\mathrm{tr}\left[ \boldsymbol{K} \right]=\sum_{i} k_{ii}$, and $\mathrm{tr}\left[ \boldsymbol{K}^{2} \right]=\sum_{ij} k_{ij}^{2}$ have been computed by iteratively loading columns (or block columns) of $\boldsymbol{K}$ into the memory, all quantities in the linear system can be computed without manipulating any $N\times N$ matrix. In particular, the residual forming matrix $\boldsymbol{P}_{\boldsymbol{0}}$ does not need to be explicitly computed. This makes the moment-matching algorithm computationally and memory efficient even if the sample size is very large.
