# Supplementary figures and images for: Phenome-wide heritability analysis of the UK Biobank
Source: PLoS Genet. 2017 Apr 7;13(4):e1006711. doi: 10.1371/journal.pgen.1006711 (PMC5400281; doi:10.1371/journal.pgen.1006711)

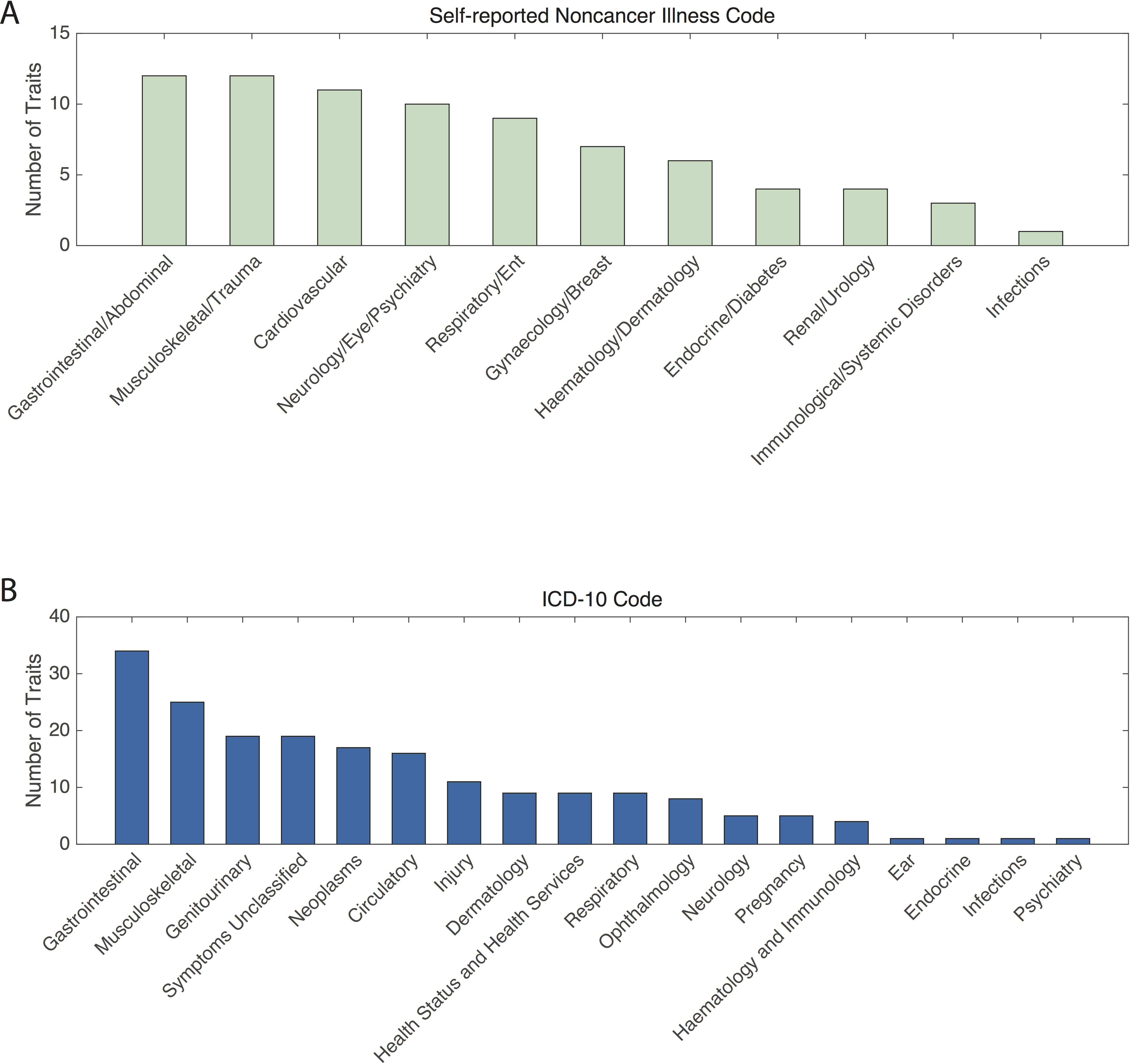

Supplement: S1 Fig — (A) A breakdown of the 79 self-reported non-cancer illness codes into different functional domains; (B) A breakdown of the 194 ICD-10 codes into different functional domains. (TIFF) [file pgen.1006711.s006.tiff]

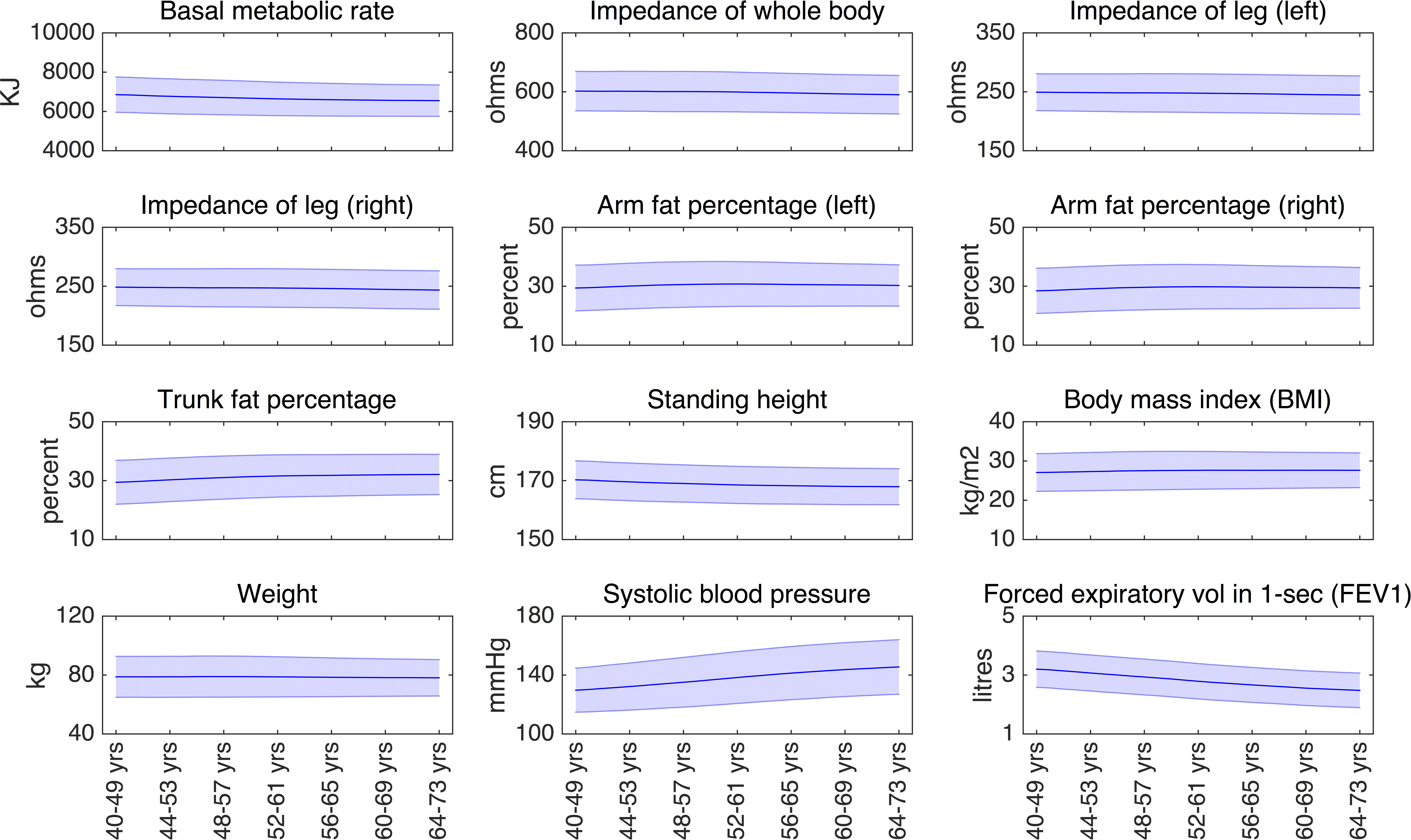

Supplement: S2 Fig — (TIFF) [file pgen.1006711.s007.tiff]

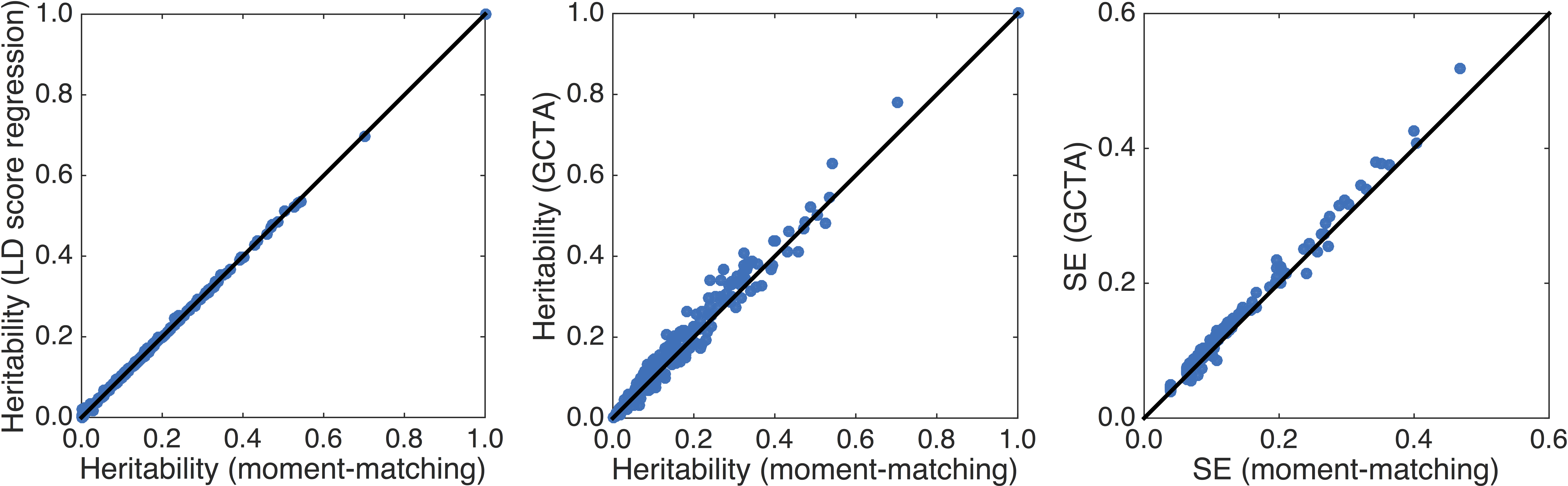

Supplement: S3 Fig — (TIFF) [file pgen.1006711.s008.tiff]

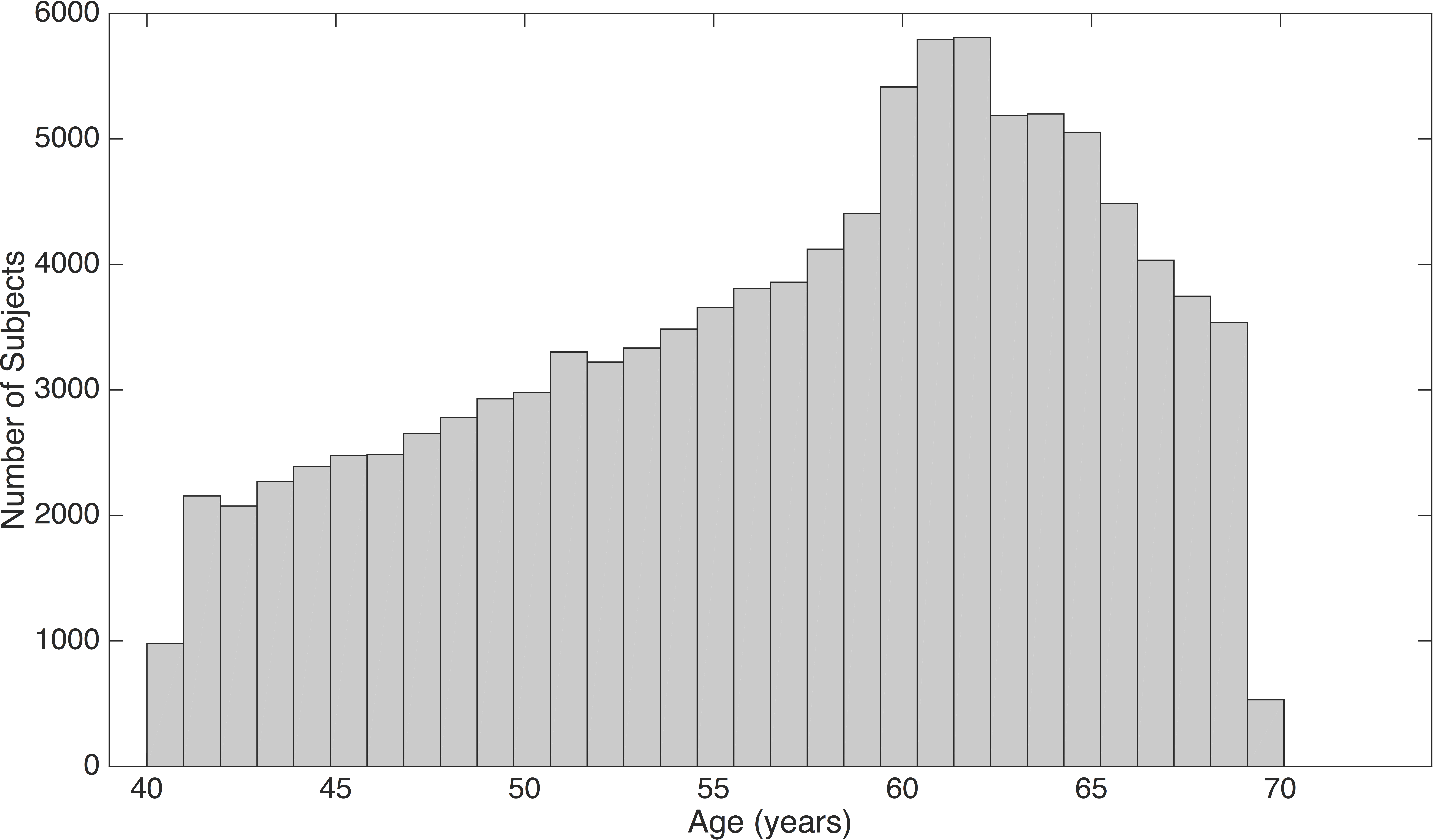

Supplement: S4 Fig — (TIFF) [file pgen.1006711.s009.tiff]
